# Semi Mature Blood Dendritic Cells Exist in Patients with Ductal Pancreatic Adenocarcinoma Owing to Inflammatory Factors Released from the Tumor

**DOI:** 10.1371/journal.pone.0013441

**Published:** 2010-10-15

**Authors:** Vegard Tjomsland, Anna Spångeus, Per Sandström, Kurt Borch, Davorka Messmer, Marie Larsson

**Affiliations:** 1 Division of Molecular Virology, Department of Clinical and Experimental Medicine, Linköping University, Linköping, Sweden; 2 Division of Internal Medicine, Department of Endocrinology, Linköping University, Linköping, Sweden; 3 Department of Medical and Health Science, Linköping University, Linköping, Sweden; 4 Division of Surgery, Linköping University, Linköping, Sweden; 5 Moores Cancer Center, University of California San Diego, La Jolla, California, United States of America; Technische Universität München, Germany

## Abstract

**Background:**

Much evidence exists regarding the fact that blood DCs, both myeloid DCs (MDCs) and plasmacytoid DCs (PDCs), are negatively affected in different types of cancer, with both reduced numbers and impaired functionality. Functional impairment of DCs in patients with pancreatic ductal adenocarcinoma (PDAC), may contribute to the poor clinical outcome. The aim of this study was to examine the effects PDAC had on blood DCs and elucidate the underlying mechanism responsible for the DC impairment.

**Methodology/Principal Findings:**

We examined the systemic influence PDAC exerted on blood DCs by ex vivo measuring numerous activation and maturation markers expressed on these cells. Furthermore, the effect patient plasma and the inflammatory factors CXCL8 and PGE_2_ had on purified MDCs and PDCs from healthy donors was assessed and compared to the DCs existing in PDAC patients. We found a partial maturation of the blood MDCs and PDCs in PDAC patients with significantly enhanced expression of CD83, CD40, B7H3, PDL-1, CCR6, and CCR7 and decreased expression of ICOSL, and DCIR. These changes lead to impairment in their immunostimulatory function. Furthermore, chronic pancreatitis gave rise to DCs with similar semi-mature phenotype as seen in PDAC. Low expression of ICOSL was associated with poor prognosis. We found that the mechanism underlying this semi-maturation of DCs was inflammatory factors existing in the PDAC patients' plasma. Of note, PGE_2_, which is elevated PDAC patient plasma, was one contributing factor to the changes seen in MDCs and PDCs phenotype.

**Conclusion/Significance:**

Our findings point to a role for the systemic inflammation in transforming blood MDCs and PDCs into semi-mature cells in PDAC patients and we show a correlation between maturation status and clinical outcome. Thus, means to preserve a functional blood DC compartment in PDAC patients by diminishing the inflammation could facilitate their ability to control the disease and improve survival.

## Introduction

Pancreatic duct adenocarcinoma (PDAC) is a lethal human cancer, with a five year survival rate of less than 5% [1], [2]. Even if PDAC is only the 10th most common cancer, the grim prognosis makes it the number four when it comes to cancer mortality [2], [3], [4]. No efficient treatment exists currently except for surgical resection, which only has a minor impact on the long term survival rate [5]. Consequently, it is of great importance to acquire a deeper knowledge about the development and progression of PDAC in order to develop new treatment strategies for this aggressive cancer.

Cancer progression and chronic infectious diseases are associated with decreased levels of blood DCs [6], [7], [8], [9], [10], [11], [12], [13], [14]. DCs are potent antigen-presenting cells that sense the presence of pathogens and serve as a link between the innate and adaptive immune system. DCs exist in tissues and blood in an immature state, but when encountering invading microbes, microbial antigens, or upon exposure to pro-inflammatory cytokines, these cells undergo a tightly regulated maturation process [15].

Peripheral blood contains two major subsets of DCs, the myeloid DCs (MDCs) and the plasmacytoid DCs (PDCs). Both MDCs and PDCs are capable of migrating to sites of inflammation where they sample antigens before migrating to the regional lymphoid tissues to mount pathogen or tumor specific immune responses. PDCs migrate from the bone marrow to the peripheral blood, but in contrast to MDCs, they relocate directly from the blood into secondary lymphoid tissue without encountering antigens and are the main producer of IFN-α in the body when activated by pathogens, especially viruses [16], [17]. DC maturation is a tightly controlled process that ensures that these potent activators of innate and adaptive immune responses do not cause autoimmunity or overreact to pathogens. When MDCs and PDCs undergo phenotypic maturation certain factors, for instance CD83, CD40, HLA DR, B7H3 (CD276) and CCR7 are upregulated, whereas DCIR, ICOSL (CD275) [18], and several tissue retaining chemokine receptors (CCR1, CCR2, CCR3, CCR5 and, CCR8) are down modulated and as a consequence the DCs will migrate to the local lymphatic tissue [19], [20], [21].

Many types of cancer, e.g. pancreatic, breast, prostate, and leukemia are associated with impaired function and reduced numbers of DCs [6], [7], [8], [9], [10], [11], [14]. Of note, we recently showed that the levels of blood DCs correlated positively with the survival of PDAC patients [14]. In breast cancer, blood DC exhibit an altered phenotype with increased level of CD83 and this correlated with disease severity [22]. Furthermore, impaired expression of CD80, CD86, and HLA DR by blood DC in patients with breast cancer and hepatocellular carcinoma, may have contributed to their decreased immunostimulatory capacity [22], [23]. The impairment in T cell activation was also an attribute for blood MDCs in PDAC [6], [24]. This imbalance in the pool of blood DCs is also observed in some chronic viral infections, autoimmune diseases, and inflammatory skin disease [12], [25], [26], [27]. For instance, partial mature DCs, i.e. semi mature DCs, have been shown to exist in HIV-1 and hepatitis C virus infected individuals [13], [28]. Increased expression of costimulatory molecules, CD40 and CD86 [28] and changes in chemokine receptor expression was evident ex vivo on DCs isolated from HIV-1 infected individuals [29]. These changes should lead to impaired DC responses.

The connection between these different medical conditions is some degree of chronic inflammation. Patients with PDAC exhibit elevated levels of circulating epithelial growth factor (EGF), IL-6, CXCL8, PGE_2_, IL-10, and IL-1RA [14], [30], [31], [32]. In accordance to these findings, high levels of several inflammatory factors including CXCL8, COX-2, and IL-6 were detected in PDAC tissues [33], indicating that the tumor tissue provides the blood with inflammatory factors.

In the present study, we investigated how the presence of PDAC altered the blood MDCs and PDCs phenotypically and functionally and the underlying mechanism/s behind the impairment of these important immune cells. Furthermore, we assessed correlations between the blood DCs expression of maturation, costimulatory, and migratory receptors with the survival of the PDAC patients. We found that the PDAC exerted systemic effects on the MDCs and PDCs, resulting in reduced numbers of DCs and creation of semi mature MDCs and PDCs with impaired T cell stimulatory ability. Furthermore, low expression of ICOSL and CCR2 by blood DCs were associated with poor clinical outcome. Of note, chronic pancreatitis induced similar MDC and PDC phenotypes as seen for PDAC, indicating that the inflammation was responsible for the semi maturation. The partial activation of MDCs and PDCs could be accomplished by exposing these cell types derived from healthy donors to recombinant PGE_2_ or to plasma from PDAC patients. These findings indicate that tumor associated inflammation does not only directly support the tumorigenesis, e.g. by releasing growth stimulatory factors, but also indirectly by impairing the ability of DCs to activate an immune response directed against the tumor.

## Results

### Blood DC levels were decreased in patients with PDAC

**Figure 1:**
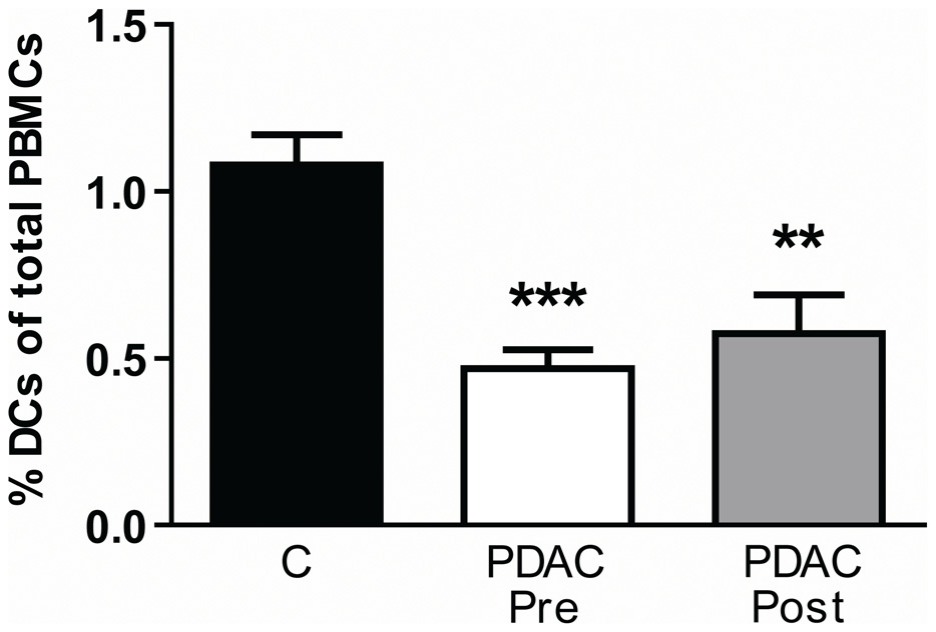
Decreased levels of MDCs and PDCs in patients with PDAC. PBMCs isolated from individuals with pancreatic duct adenocarcinoma (PDAC) (1 week pre and 12 weeks post surgery) and healthy age matched volunteers were analyzed for DC levels by flow cytometry. The PBMCs were stained with Linage cocktail, HLA DR, CD11c, and CD123 direct conjugated mabs to distinguish all DC subsets from the rest of the cells. The DC levels were calculated from the total amount of PBMCs and compared between the different groups using nonparametric Wilcoxon signed rank test used for paired data and Mann–Whitney test for calculation of p values. Statistically significant differences between individuals with PDAC and healthy controls are indicated as; * = p<0.05, ** = p<0.005, *** = p<0.001.

Blood DCs in many cancers display both impaired numbers and functionality [6], [7], [8], [9], [10], [11], [24], [34]. The levels of total blood DCs, i.e. MDCs and PDCs, in individuals with PDACs were compared to age match controls. The frequency of DCs in blood was measured as the percentage of total PBMCs. We found a significant decrease in blood DCs, both pre (median 0.2%) and post surgery (median 0.6%) compared to healthy controls (median 1.0%) (**Figure 1**). Our age matched controls had equivalent levels of MDCs and PDCs as documented previously for this age group [35], [36].

### MDCs and PDCs from PDAC patients had increased levels of the maturation marker CD83 and the costimulatory molecule CD40

**Figure 2:**
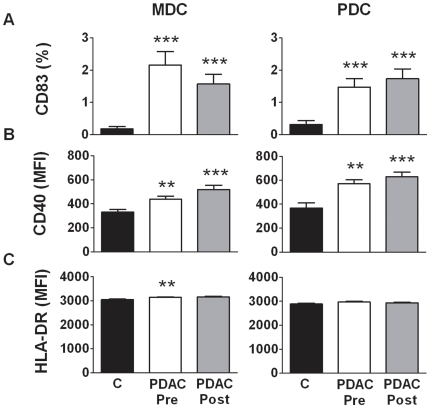
Increased levels of activation markers CD83 and CD40 on blood DCs in patients with PDAC. Blood MDCs (HLA DR^+^CD11c^+^Lin^−^) and PDCs (HLA DR^+^CD123^+^Lin^−^) were detected in PBMCs obtained from PDAC patients pre (1 week) and post (12 weeks) surgery and healthy age matched individuals. Both DC subsets were investigated for the expression of the maturation marker CD83 (A), co-stimulatory molecule CD40 (B), and HLA DR (C). Mean fluorescence intensity (MFI) and percentage (%) of positive MDCs and PDCs and compared between the different groups using nonparametric Wilcoxon signed rank test used for paired data and Mann–Whitney test for calculation of p values. Statistically significant differences between individuals with PDAC and healthy controls are indicated as; * = p<0.05, ** = p<0.005, *** = p<0.001.

Under normal conditions MDCs and PDCs exist in an immature state in the peripheral blood and are only activated when exposed to pathogens or inflammation after which they gain the ability to enter tissue and lymphoid organs [37]. We assessed if levels of the maturation marker CD83, costimulatory molecule CD40, and HLA DR on blood MDCs and PDCs were affected by the presence of PDAC. CD83 is a glycoprotein that is upregulated together with costimulatory molecules, such as CD40 and CD86, thus suggesting a role in the induction of immune responses [38]. We observed that CD83 was significantly elevated both in MDCs (median 1.8%) and PDCs (median 2.8%) in patients with PDAC compared to control MDCs (median 0%) and PDCs (median 0%) (**Figure 2A**). Of note, elevated levels of CD83 positive MDCs and PDCs were also observed in billary duct adenocarcinoma, ampullary carcinoma, and endocrine carcinoma, all cancers in or in connection to the pancreas (data not shown). The removal of the tumor had no or very low effect on CD83 levels when measured ∼12 weeks after surgery, as the CD83 expression remained higher on MDCs (median 0.9%) and PDCs (median 1.5%) compared to controls (**Figure 2A**). We also measured the level of CD40, i.e. a costimulatory molecule important for the activation and survival of DCs and found significantly increased levels of CD40 on MDCs and PDCs both before (median 432; median 584) and after tumor resection (median 524, median 656), compared to those expressed on control MDCs (median 322) and PDCs (median 349) (**Figure 2B**). Furthermore, the expression of HLA DR was only affected on MDCs prior (median 3162) to tumor removal compared to healthy controls (median 3046) (**Figure 2C**). No effect was seen on PDCs.

### MDCs and PDCs in PDAC patients had decreased expression of the C-type lectin DCIR

**Figure 3:**
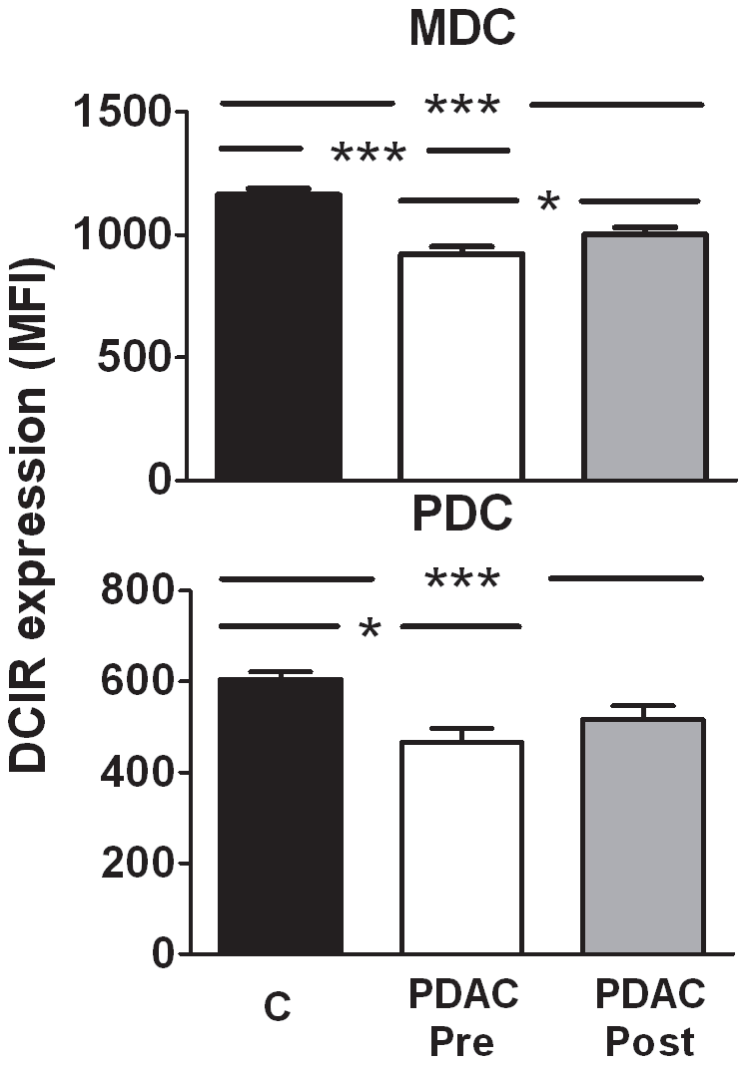
The C type lectin DCIR was down regulated on MDCs and PDCs in PDAC patients. Dendritic cell subsets, MDCs (HLA DR^+^CD11c^+^Lin^−^) and PDCs (HLA DR^+^CD123^+^Lin^−^), were distinguished form PBMCs obtained from PDAC patients pre (1 week) and post (12 weeks) surgery and age matched control individuals by flow cytometry. Changes in DC phenotype were detected using direct conjugated antibody for dendritic cell immunoreceptor (DCIR). Mean fluorescence intensity (MFI) values from the patients included in the different groups were compared using nonparametric Wilcoxon signed rank test, used for paired data and Mann–Whitney test for calculation of p values. Statistically significant differences between individuals with PDAC and healthy controls are indicated as; * = p<0.05, ** = p<0.005, *** = p<0.001.

We observed that DCIR, a C-type lectin that is down modulated upon DC maturation [18], was significantly decreased in MDCs and PDCs both pre (median 939, median 453) and post surgery (median 993 median 509) in patients with PDAC as compared to controls (median 1177, median 612). However, a tendency of recovery in DCIR levels was found post surgery in the myeloid but not plasmacytoid DCs (**Figure 3**).

### MDCs and PDCs in PDAC patients exhibited an activated profile of B7-family members

**Figure 4:**
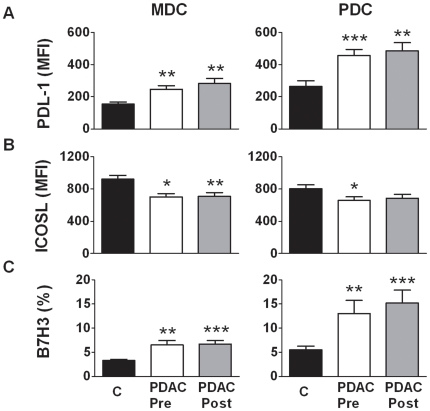
Increased B7 family expression profile on MDCs and PDCs in patients with PDAC. Blood MDCs (HLA DR^+^CD11c^+^Lin^−^) and PDCs (HLA DR^+^CD123^+^Lin^−^) were detected in PBMCs obtained from PDAC patients pre (1 week) and post (12 weeks) surgery and healthy age matched individuals. Both DC subsets were investigated for the expression of co-stimulatory molecules from B7 family using direct conjugated antibodies against PDL-1 (A), ICOSL (B) and B7H3 (C) and analyzed using 8 color flow cytometry. Mean fluorescence intensity (MFI) values or present positive MDCs and PDCs were compared between the different groups using nonparametric Wilcoxon signed rank test used for paired data and Mann–Whitney test for calculation of p values. Statistically significant differences between individuals with PDAC and healthy controls are indicated as; * = p<0.05, ** = p<0.005, *** = p<0.001.

We examined the expression profile of members of the B7-family, i.e. PDL-2 (B7DC: CD273), PDL-1 (CD274:B7H1), B7H3 (CD276), ICOSL (CD275) on blood MDCs and PDCs from PDAC patients. The levels of PDL-1 and B7H3 were increased on MDCs pre surgery (median 245, median 5.1) and post surgery (median 252, median 5.2) compared to controls (median 164, median 3.3). Similar results were found for PDCs pre (median 482, median 9.8) and post surgery (median 438, median 12.2) in PDAC patients compared to the levels in healthy controls (PDL-1: median 214, B7H3: median 4.0) (**Figure 4A and C**). ICOSL levels were significantly decreased on MDCs pre and post surgery (median 641, median 684) compared to the control group (median 993). PDCs displayed only a significant decrease pre surgery (median 598) compared to controls (median 844) (**Figure 4B**). We could not detect ex vivo expression of B7DC on MDCs and PDCs (**Data not shown**). The upregulation of PDL-1 and down modulation of ICOSL fits the profile of activated DCs, albeit not a complete activation as they do not reach the levels on fully mature DC. Of note, the increased expression of B7H3 did not correlate with activated matured MDCs or PDCs as this costimulatory molecule is normally down regulated upon maturation [39].

### DCs from PDAC patients had increased expression of chemokine receptors

**Figure 5:**
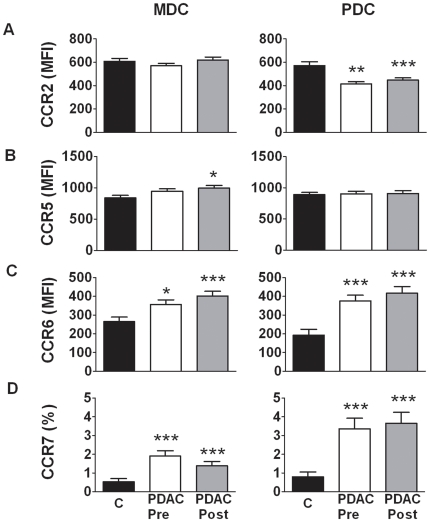
Chemokine receptor expression profile on MDC and PDCs in patients with PDAC. Blood MDCs (HLA DR^+^CD11c^+^Lin^−^) and PDCs (HLA DR^+^CD123^+^Lin^−^) from PDAC patients pre (one week) and post (12 weeks) surgery were compared to age matched controls. The DC subsets from each group were stained using direct conjugated antibodies against CCR2 (A), CCR5 (B), CCR6 (C), and CCR7 (D) and detected by multi-color flow cytometry. Mean fluorescence intensity (MFI) values or the amount positive MDCs and PDCs in percentage were compared between the different groups using nonparametric Wilcoxon signed rank test used for paired data and Mann–Whitney test for calculation of p values. Statistically significant differences between individuals with PDAC and healthy controls are indicated as; * = p<0.05, ** = p<0.005, *** = p<0.001.

Blood PDCs express many different chemokine receptors including CCR2, CCR5, CCR6, CCR7, CXCR3, and CXCR4 [40], whereas MDC express CCR1, CCR2, CCR3, CCR5, CCR6, CCR7 and CCR8 [19], [20], [21]. Some of these chemokine receptors have the ability to retain DCs in tissue, whereas others are involved in the migration of DCs into other organs such as CCR7 into lymphoid tissue. We measured CCR2, CCR5, CCR6, and CCR7, on DCs before and after surgical removal of the pancreatic tumor mass (**Figure 5A–D**). Significantly increased levels of CCR6 were found on MDCs pre and post surgery (pre: median 380, post: median 398) compared to healthy individuals (median 255) (**Figure 5C**), and for CCR7 (pre: median 1.6%, post: median 1.6%, control: median 0%) (**Figure 5D**) but not for CCR2 (**Figure 5A**). CCR5 expression by MDCs was affected only post surgery (post: median 980, control: median 848) (**Figure 5B**). The profile for PDCs, both pre and post surgery, showed increased levels for CCR6 (pre: median 405, post: median 440, control: median 166) (**Figure 5C**) and CCR7 (pre: median 3.2, post: median 3.2, control: median 0) (**Figure 5D**), but no effect was seen for CCR5 (**Figure 5B**). Although MDCs displayed unchanged levels of CCR2, PDCs expressed decreased levels of CCR2 both pre and post surgery (pre: median 437, post: median 441) compared to controls (median 602) (**Figure 5A**).

### Chronic pancreatitis produced semi-mature MDCs and PDCs similar to the cells found in PDAC patients

**Figure 6:**
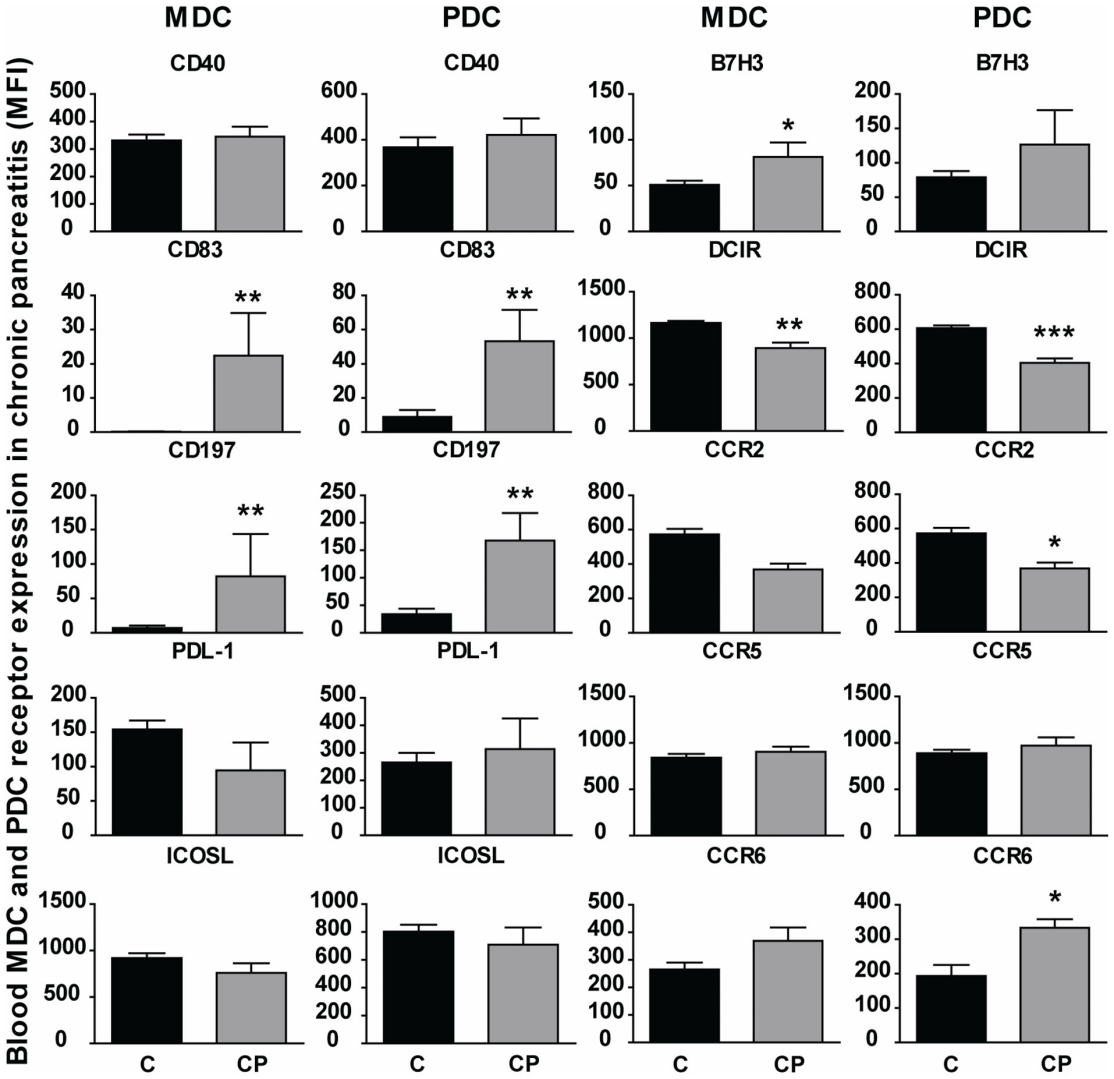
Blood DCs from chronic pancreatitis patients exhibited a similar semi-mature phenotype seen in PDAC patients. Dendritic cell subsets, MDCs (HLA DR^+^CD11c^+^Lin^−^) and PDCs (HLA DR^+^CD123^+^Lin^−^), were distinguished form PBMCs obtained from chronic pancreatitis (CP) patients (N = 5) and age matched control individuals by flow cytometry. Changes in DC phenotype were detected using direct conjugated antibody for CD40, CD83, CCR2, CCR6, CCR5, CCR7, PDL-1, ICOSL, DCIR, and B7H3. Mean fluorescence intensity (MFI) values from the patients included in the different groups were compared using nonparametric Wilcoxon signed rank test, used for paired data and Mann–Whitney test for calculation of p values. Statistically significant differences between individuals with CP and healthy controls are indicated as; * = p<0.05, ** = p<0.005, *** = p<0.001.

To explore if the if chronic inflammation of pancreas was responsible for the induction of semi-mature DCs in PDAC did we examine the phenotype of blood MDCs and PDCs ex vivo from patients with chronic pancreatitis (CP). The phenotypic profiles for MDCs and PDCs in CP and PDAC were the same for many of the surface molecules examined. MDCs and PDCs had significantly increased levels of CD83 (MDC; p = 0.002 and PDC; p = 0.002), CCR7 (MDC; p = 0.002 and PDC; p = 0.004), and significantly decreased levels of DCIR, which matched the findings in PDAC patients (**Figure 6**). The expression of CCR2 and CCR6 by PDCs and MDCs did also match the profile found in PDAC, but only CCR2 (p = 0.002) and CCR6 (p = 0.045) on PDCs were significantly affected (**Figure 6**). Expression of the negative costimulatory molecule PDL-1 by MDCs differed between PDAC and CP with increased expression on cells from PDAC but not from CP (**Figure 4A** **and** **6**). The slight difference in profile could be due to the factors provided by the tumor or differences in levels of inflammatory factors between these two diseases.

### Blood MDC and PDC in PDAC patients had impaired ability to activate T cells

**Figure 7:**
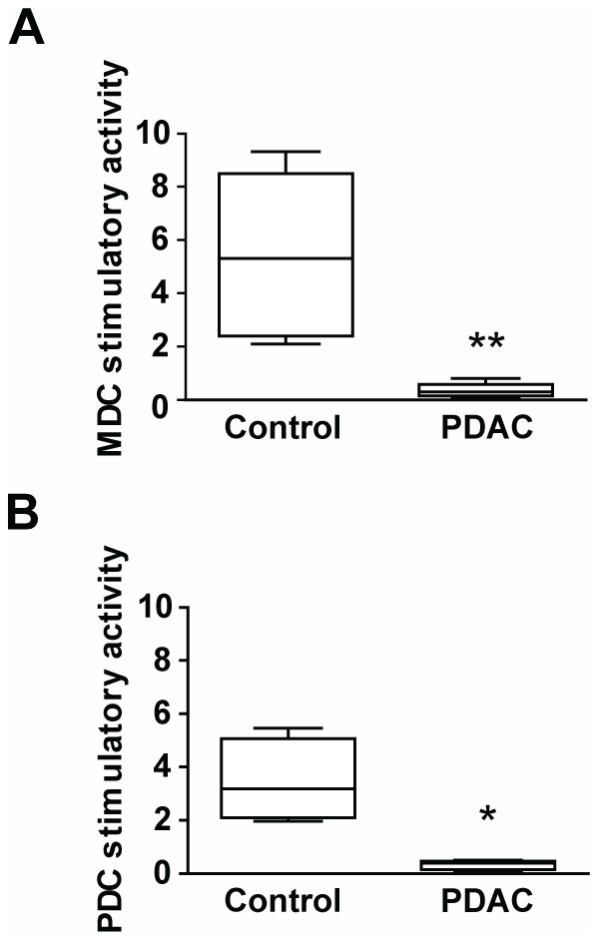
MDC and PDC in PDAC patients have impaired immunostimulatory function. **A**–**B**) Immunostimulatory capacity of blood DC tested by mixed leukocyte reaction (MLR). MDCs (**A**) and PDCs (**B**) purified from PDAC patients (N = 6) or healthy controls (N = 6) were cocultured with allogeneic T cells for 5 days and T cell proliferation assessed by ^3^H-Thymine incorporation. Comparison of stimulatory capacity was assessed by dividing the counts per minute (CPM) of patients with the mean CPM of controls and the CPM of the controls with the mean CPM of the patients for each plate analyzed. Statistical significance was determined by Mann–Whitney test for calculation of p values. * = p<0.05, ** = p<0.005.

Functionality of the blood MDC and PDC in PDAC patients (N = 6) was examined by assessing their ability to activate allogeneic T cells in mixed lymphocyte reaction (MLR). Both MDCs (p = 0.004) and PDCs (p = 0.029) from PDAC patients had significantly impaired ability to induce T cell proliferation compared to healthy controls (**Figure 7**) The MDCs had a 2–8 fold decreased stimulation capacity compared to DCs from healthy controls (**Figure 7**), confirming previous findings for PDAC patients [6], [24]. The PDCs had a 2–5 fold decreased immunostimulatory activity (**Figure 7**) and this has not been shown previously for PDAC patients.

### MDC and PDC levels of ICOSL and CCR2 pre surgery, correlated with the PDAC patient's clinical outcome

**Figure 8:**
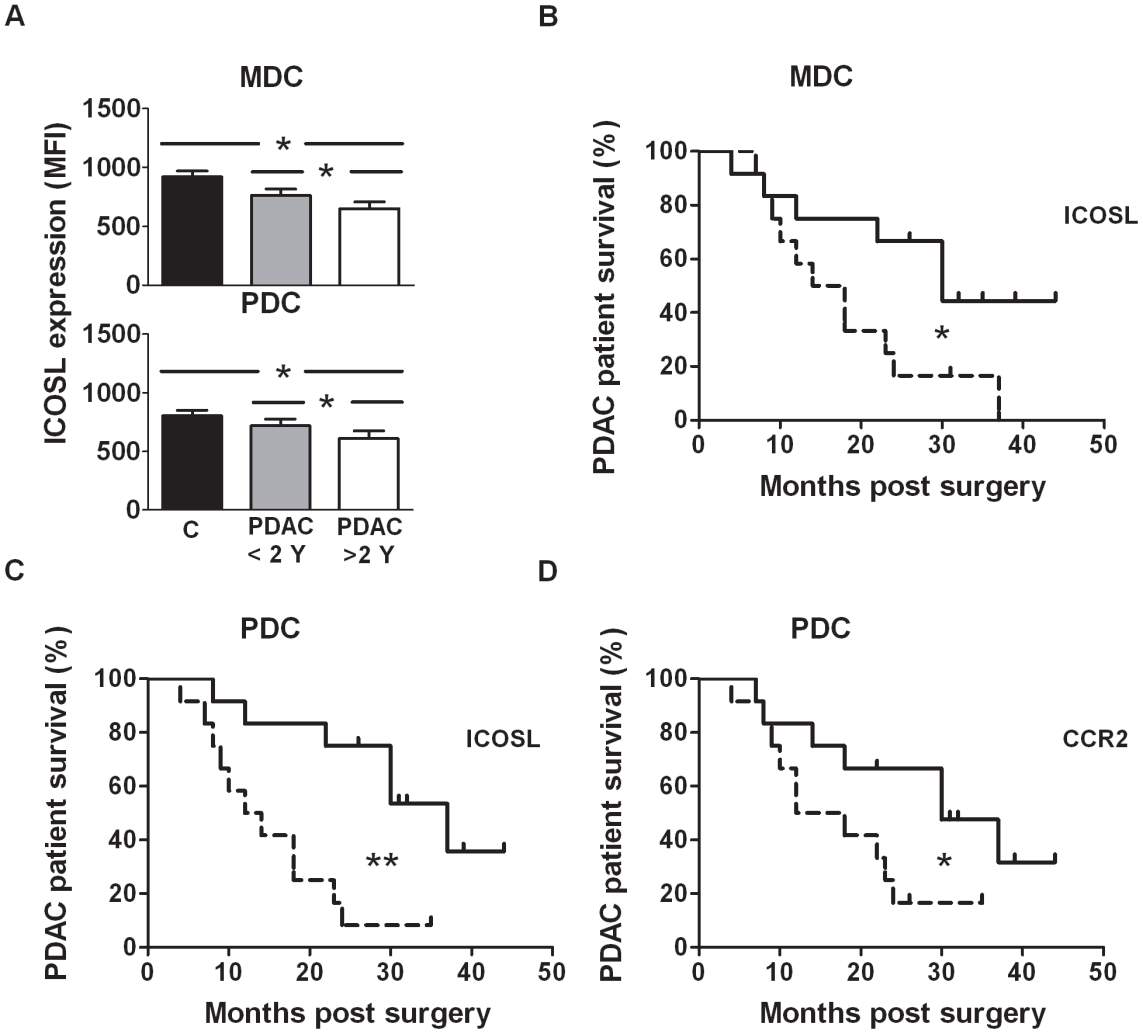
Low expression levels of ICOSL and CCR2 correlated to poor prognosis in PDAC patients. Median fluorescence intensity (MFI) levels for ICOSL on MDCs and PDCs pre surgery were correlated to patient survival time. (**A**) The patients were divided into two groups, one group consisting of patients surviving more than two years (N = 11) and the second group of patients surviving less than two years (N = 13). Comparison of the survival between the PDAC patient group with the lowest (scattered line) (N = 12) and highest (N = 12) mean fluorescence intensity (MFI) levels of ICOSL for (**B**) MDCs (≤641MFI and >641MFI), (**C**) PDCs (≤598MFI and >598MFI) and MFI levels of CCR2 for (**D**) PDCs (≤601.5MFI and >601.5MFI) pre surgery. Long-rank (Mantel-Cox) test was used for calculation of p values. * = p<0.05, ** = p<0.005.

To evaluate if the change in MDC and PDC surface markers could predict patient survival, all markers examined were tested against patient survival. The PDAC patients with a survival of more than two years had significantly higher levels of ICOSL on both MDCs (p = 0.031) and PDCs (p = 0.043) than patients with a survival of less than 2 years (**Figure 8A**). The patients were further ranked as high expression (>median) or low expression (<median) of each factor and correlated to the survival. Patients with high levels of ICOSL on the MDCs (range 641–1141) or PDCs (range 598–1212) pre surgery had significantly longer survival time (MDCs: p = 0.037, PDCs: p = 0.0025) than patients expressing low levels (MDC: range 481–641, PDC: range 410–598). Moreover, patients with high CCR2 levels on their PDCs (range 437–581) had a better clinical outcome (p = 0.048) than patients with low levels (range 206–437) (**Figure 8B–D**). Decreased levels of ICOSL and CCR2 are typical features of mature DCs and our results indicate that the PDAC patients with the best clinical outcome also had DCs with a less activated phenotype.

### The maturation profile for MDCs and PDCs after stimulation with TLR ligands in vitro correlated with the semi mature DCs in PDAC patients

**Figure 9:**
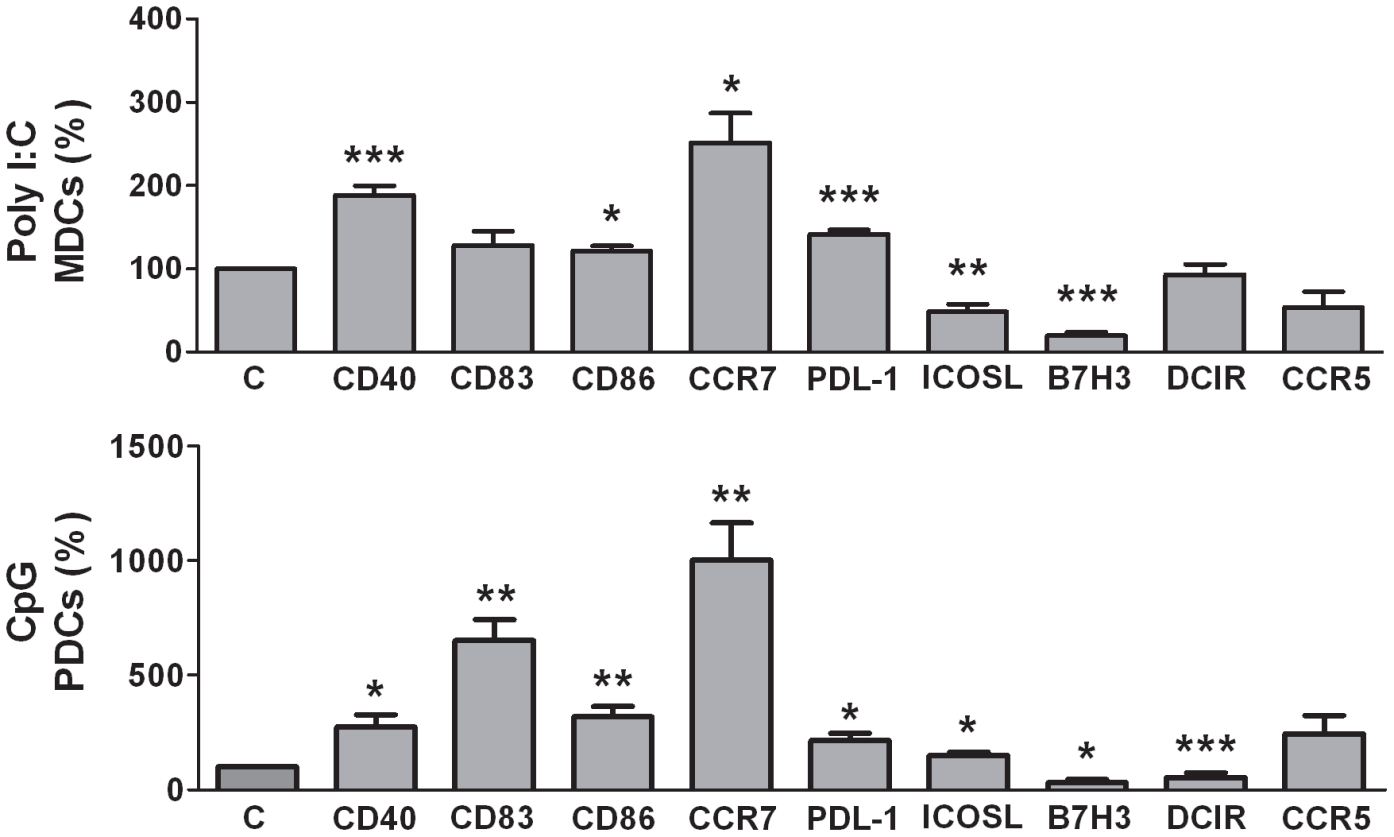
Maturation profile of MDCs and PDCs after exposure to TLR ligand. MDC and PDC populations were sorted on a FACS ARIA cell sorter from T cells, B cells, NK cells and monocytes depleted PBMCs using lineage cocktail (Alexa Fluor 700), HLA DR (APC-Alexa Fluor 750), CD11c (PE-Cy7) and CD123 (PerCP-Cy5.5) mabs. The DC subsets were cultured in separate wells for 24 hours in 1% plasma medium with or without the presence of TLR3 ligand (Poly I:C) for MDCs or TLR9 ligand (C CpG) for PDCs. The cells were harvested and stained using direct conjugated antibodies against CD40, CD83, CD86, CCR5, CCR7, PDL-1, ICOSL, DCIR, and B7H3 and analyzed using multi-color flow cytometry. The data were normalized to the control medium and paired t test was performed for calculation of p values. Statistically significant differences between individuals with PDAC and healthy controls are indicated as; * = p<0.05, ** = p<0.005, *** = p<0.001.

TLR ligation induced a potent DC maturation and here we correlated the ex vivo maturation profile seen for MDCs and PDCs in PDAC patients with the one induced by TLR activation of blood MDCs and PDCs purified from healthy volunteers. The purified MDCs and PDCs were matured with the TLR3 ligand Poly I:C and TLR9 ligand CpG, respectively, and assessed for CD40, CD83, CD86, PDL-1, ICOSL, B7H3, DCIR, CCR5, and CCR7 expression levels. The TLR3 ligation induced a MDC maturation profile with upregulation of the markers; CD40, CD83, CD86, CCR7, PDL-1, and downregulation of ICOSL, B7H3, and CCR5 (**Figure 9**). The TLR9 ligation on PDCs induced a mature phenotype with upregulation of CD40, CD83, CD86, CCR7, PDL-1, ICOSL and CCR5 and decreased B7H3 and DCIR (**Figure 9**). Of note, we did see a down modulation of CCR2, and CCR6 expression to almost undetectable levels compared to CCR2 and CCR6 levels seen on ex vivo DCs from PDAC patients, independent of the treatment of the DCs, i.e. untreated or activated by TLR ligands. MDCs and PDCs expression of CCR5 was also decreased to very low levels and this decease in chemokine receptors seemed to be due to the 24 h in vitro culture. Similar down regulation of CCR2 has been documented for monocytes when cultured overnight [41]. The phenotype of ex vivo semi mature MDC and PDCs from PDAC patients matched the profile of in vitro cultured and TLR ligand matured blood DCs, with the exception for a higher B7H3 expression. Noteworthy, the ex vivo DCs levels of activation and maturation markers did not reach the levels seen for fully matured DCs, i.e. they had a semi mature phenotype.

### Semi maturation of blood DCs was induced by factors present in the PDAC patients' plasma

**Figure 10:**
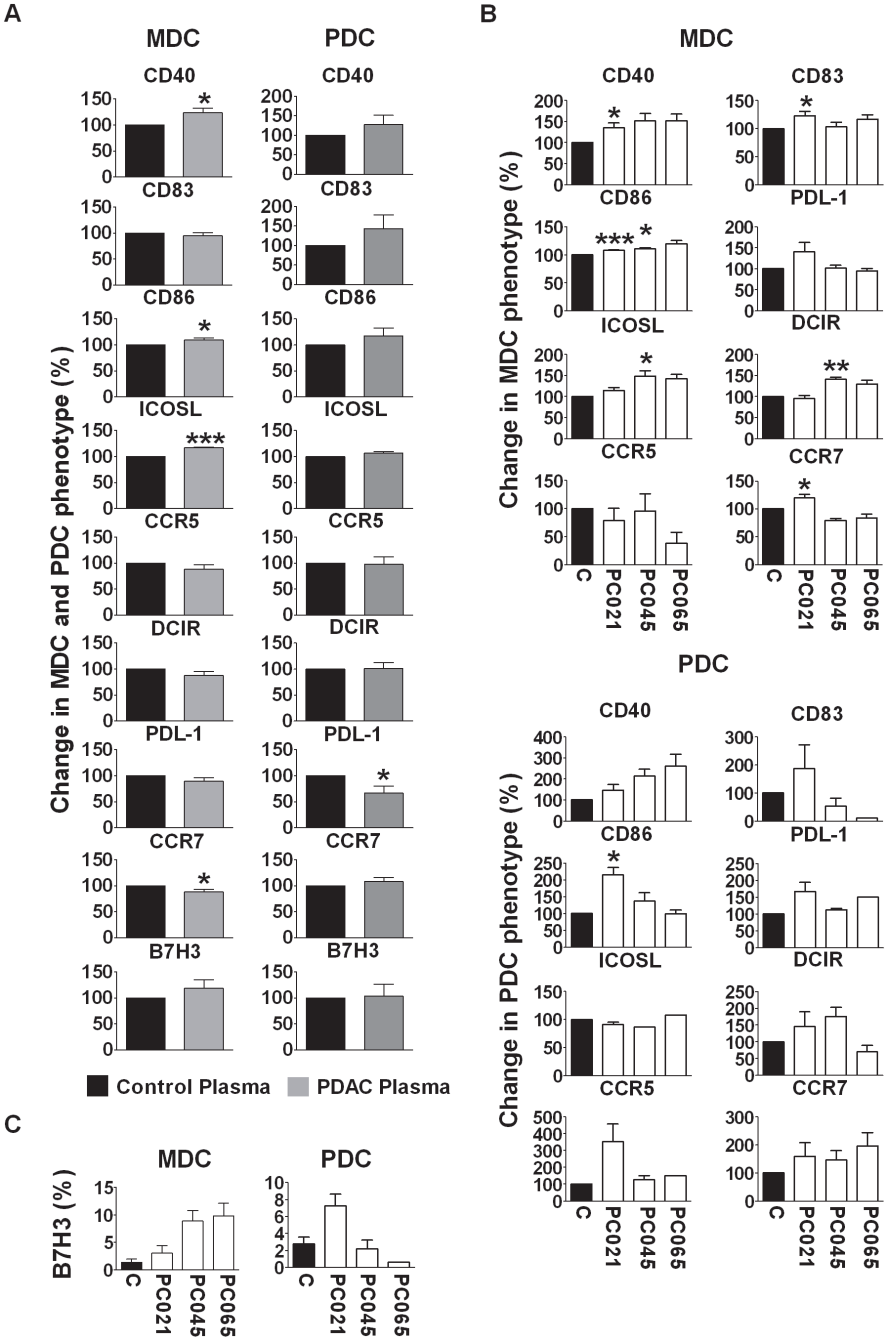
Exposure to plasma from PDAC patients induced activation of MDCs and PDCs from healthy individuals. MDCs and PDCs from healthy blood donors were freshly isolated from PBMCs using direct conjugated antibodies followed by FACS sorting. (**A**) The DC subsets were cultured in separate wells for 24 hours in 1:4 diluted pooled human plasma medium obtained from PDAC patients or healthy controls. The cells were harvested and stained using direct conjugated antibodies against CD40, CD83, CD86, CCR5, CCR7, PDL-1, ICOSL, DCIR and B7H3 and analyzed using multi-color flow cytometry. (**B**) MDCs were cultured in separate wells for 24 hours in 25% single human plasma medium obtained from PDAC patients (PC013, PC021, PC045, or PC065) and one representative age matched healthy control (C2). The cells were harvested and labeled by direct conjugated antibodies against CD40, CD83, CD86, CCR5, CCR7, PDL-1, ICOSL, DCIR, and B7H3 (presented as non normalized data as the control plasma diminished the levels in some experiments (C) and analyzed by multi-color flow cytometry. Mean fluorescence intensity (MFI) or present positive DCs were normalized to controls and paired t-test was used for calculation of p values. Statistically significant differences between individuals with PDAC and controls are indicated as; * = p<0.05, ** = p<0.005, *** = p<0.001.

Purified MDCs and PDCs from healthy donors were incubated for 24 h with a mix of 1:4 diluted plasma from 6 PDAC patients, or from 6 age matched controls, or 1:4 diluted single donor plasma from PDAC patients or healthy donors for 24 h. The phenotypic profile was assessed for the mixed plasma and we found a correlation with the ex vivo profile of MDCs and PDCs from PDAC patients for some but far from all factors (**Figure 10A**). The combined plasma induced upregulation of CD40 (median 118, median 98), and CD86, (median 105, median 112), in both MDCs and PDCs. CD83 expression was only elevated on PDCs (median 96). The B7H3 expression showed a tendency to be increased in MDCs and PDCs (**Figure 10A**) which fits the ex vivo data (**Figure 4C**). The mix of PDAC plasmas did not have any clear effects on CCR7, PDL-1, and DCIR (**Figure 10A**). We examined the effect of single PDAC plasma from patients, PC021, PC045, and PC065 on purified blood DCs. Semi-mature profiles were seen for both MDCs and PDCs matured with PC021 plasma with significantly increased levels for CD40, CD83, CD86, and CCR7 on MDCs and enhanced levels of CD40, CD86, CD83, CCR7, and PDL-1 on PDCs (**Figure 10B**), which was a more clear activation than that achieved by pooled plasma. The profile induced by the plasma from PC045 and PC065 on MDCs and PDCs was not as clear cut with only a few of the activation/maturation factors such as CD40, and CD86 upregulated on both sets and mixed effect on the other factors measured (**Figure 10B**). Despite the use of diluted (1:4) patient plasma, we were able to confirm some of the markers that were altered on blood DCs in PDAC patients.

### Inflammatory factor PGE_2_, present in the PDAC plasma, was involved in the induction of semi mature DCs

**Figure 11:**
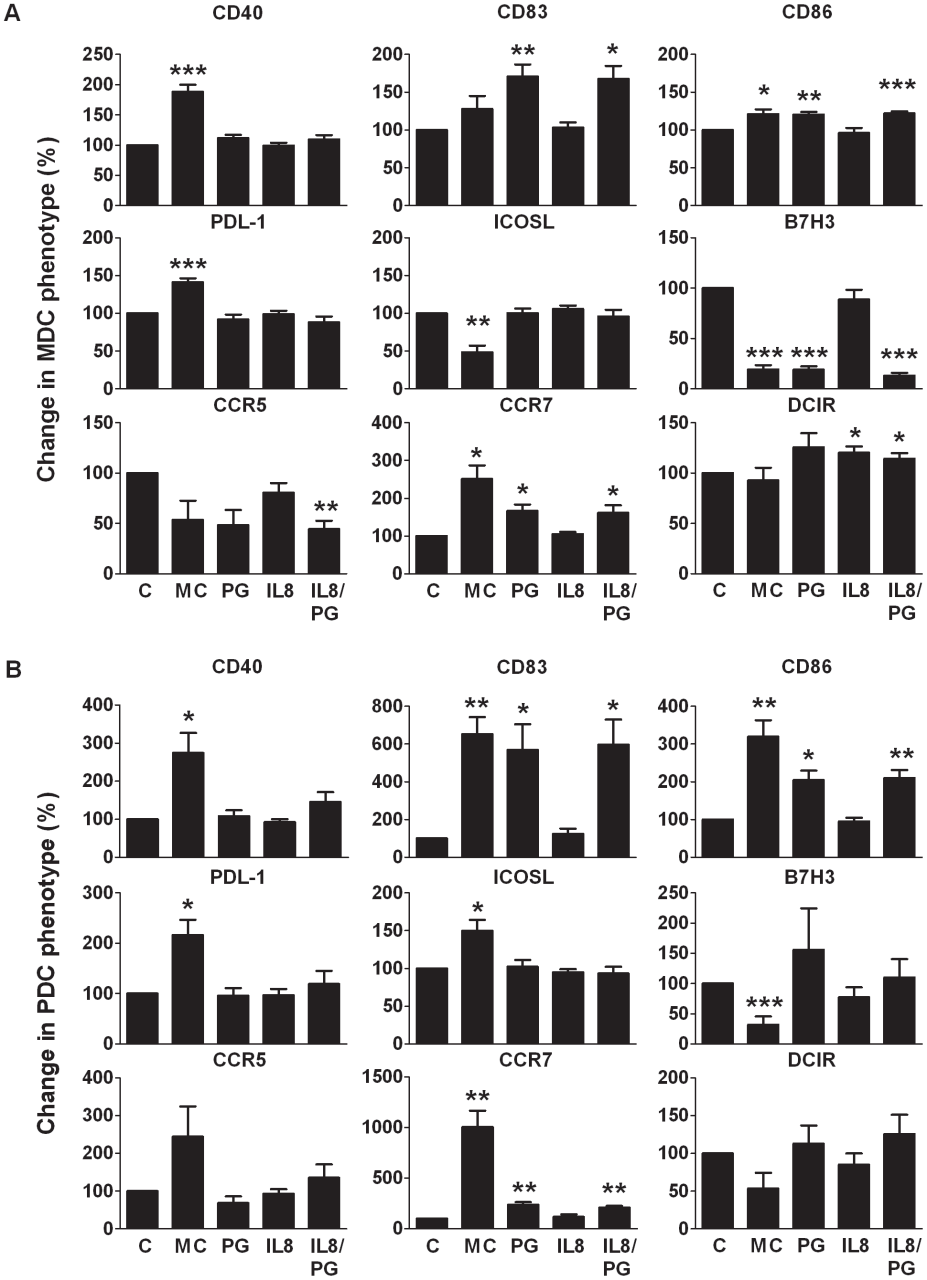
PGE_2_ exposure induced a partial activation of healthy purified MDCs and PDCs. FACS sorted MDCs and PDCs obtained from healthy donors were cultured in separate wells for 24 hours in 1% plasma medium with or without the presence of TLR3 ligand (Poly I:C) for MDCs or TLR9 ligand (C CPG) for PDCs, 10 ng/ml PGE_2_, 10 ng/ml CXCL8 (IL-8), or 10 ng/ml PGE_2_ in combination with 10 ng/ml CXCL8 (A). The MDCs (A) and PDCs (B) were harvested and labeled by direct conjugated antibodies against CD40, CD83, CD86, CCR5, CCR7, PDL-1, ICOSL, DCIR, and B7H3 and analyzed using multi-color flow cytometry. The data are normalized to the control medium and paired t test was performed for calculation of p values. Statistically significant differences between individuals with PDAC and healthy controls are indicated as; * = p<0.05, ** = p<0.005, *** = p<0.001.

We have previously assessed the inflammatory profile for PDAC patients and only CXCL8, and PGE_2_ were found to be significantly enhanced, both in plasma [14] and in the tumor tissue (**data not shown**). To establish if CXCL8 or PGE_2_, or a combination of these factors had the ability to induce similar effects on MDCs and PDCs as seen ex vivo, purified MDCs and PDCs from healthy donors were exposed to CXCL8 and PGE_2_. We found that the combination of CXCL8 and PGE_2_ had a similar effect on MDCs and PDCs expression pattern for most of these markers as seen for PGE_2_ treatment alone (**Figure 11**). PGE_2_ induced higher expression of CD83, CD86, and CCR7 on both MDCs and PDCs than accomplished by CXCL8 (**Figure 11**). CD40 expression was enhanced, though not significantly, on MDCs and PDCs by treatment with the combination of CXCL8 and PGE_2_ (**Figure 11**). The expression of B7H3 on MDCs decreased after exposure to PGE_2_, or to a combination of CXCL8 and PGE_2_, whereas expression of B7H3 on PDCs diverged between experiments (**Figure 11**). Exposing the DCs to a combination of two factors found elevated in PDAC plasma, PGE_2_ and CXCL8, created to some extent the MDC and PDC phenotype found in PDAC patients ex vivo. Our results prove that solid pancreatic tumors, including PDAC, systemically affect blood DCs.

## Discussion

We show in this study that the PDAC exerted systemic effects on the MDCs and PDCs, resulting in semi matured cells with impaired immunostimulatory function and reduction in DC numbers [14]. The partial activation of MDCs and PDCs could be accomplished by exposure of these cells, purified from healthy donors, to plasma derived from PDAC patients. Noteworthy, we also show that CXCL8 and PGE_2_, two of the inflammatory factors that are significantly enhanced in PDAC plasma, are involved in the DC activation and we speculate that multiple inflammatory factors leak out from the tumor microenvironment and exert a systemic effect on the immune cells.

Partial or incomplete activation, as seen in our study, has previously been described for DCs in several chronic infections [13], [42]. Semi mature DCs were detected in lymphoid tissues in HIV infected patients [42], and in the blood of HCV infected patients [13], [43]. The DCs in the HIV patients showed increased CD40 on MDCs and decreased CD86 on PDCs [42]. In PDAC, the phenotypic alteration seen for PDC and MDCs may result from the chronic inflammation created by the tumor both locally and systemically [14]. This is supported by the fact that these cells in chronic pancreatitis exhibited a similar phenotype as seen for PDAC. The increase of some costimulatory markers but no or low enhancement of MHC class II expression could implicate impaired MHC II antigen presenting abilities. Especially, considering the fact that the PDL-1 and B7H3 levels were increased, providing more factors that impair the ability of DCs to activate T cell responses. Evidence supporting this, is the blood DCs impaired ability to stimulate T cells shown in this study for both MDCs and PDCs and previously proven for MDCs [6]. Breast cancer is another example with decreased T cell stimulatory ability, which also exhibit decreased IL-12 production and increased IL-10 production [8].

Elevated levels of inflammatory factors have been detected in individuals with pancreatic cancer [30], [31], [32]. High serum IL-6 and IL-10 levels correlated with poor survival [31]. Over expression of CXCL8 and COX-2 mRNA was found in most of the PDAC tissues [43](data not shown). Continual production of inflammatory factors by the tumor adjacent tissues could be an explanation for the sustained negative effect exerted on DCs despite the removal of the tumor mass [14]. Of note, when the main producers of the inflammation, i.e. tumor cells and fibrotic stroma, were removed the systemic levels of CXCL8 and PGE_2_ in blood were decreased [14]. In this study we confirmed that these factors, especially PGE_2_, were involved in the partial DC maturation. This verifies the important role of inflammation in the dysfunctional immune responses seen in PDAC and other types of cancer. Of note, these factors play an essential role in creating an environment that sustains the tumor and its ability to metastasize.

Under normal conditions, MDCs and PDCs exist in peripheral blood circulation only in an immature state and should when activated leave the blood stream and enter tissues and lymphoid organs [37]. We found an increased expression of several activation markers on blood MDCs and PDCs in individuals with PDAC. This pattern correlated with semi mature DCs, as the activation level was relatively lower compared to fully matured DCs. The CD83, CD40 and CD86 are upregulated as a step in the maturation process and have a role in immune modulation [38]. Increased levels of CD40 and CD83 were observed on PDCs from HCV infected patients [13], whereas MDCs were less affected [13]. CD40 and CD83 expression were significantly elevated both on MDCs and PDCs in patients with PDAC, but did not reach the level seen in fully mature cells. Of note, a partial mature blood DC phenotype, with elevated CD83 expression, correlated with the severity of breast cancer [22].

Several inflammatory chemokine receptors were increased on the blood DCs in PDAC patients. CCR6 and CCR7 were increased on both MDCs and PDCs. CCR6 regulates the migration and recruitment of DCs and T cells during inflammation, whereas CCR7 controls the migration of DCs and T cells to lymphoid organs due to production of CCL19 and CCL21 at these sites [44]. Elevated levels of CCR6 on blood PDCs were detected in melanoma patients [45] and this concur with our findings. Furthermore, these PDCs migrated toward CCL20 and could be found in melanoma lesions [45]. To speculate, the CCR6 high PDCs in our system might migrate into the CCL20 positive pancreatic tumor microenvironment [33]. For PDCs, CCR2 was significantly decreased, whereas the MDCs were unaffected. CCR2 is normally involved in the infiltration of monocytes in inflammatory diseases such as rheumatoid arthritis and its ligands include CCL2, CCL7, and CCL8.

Whether the DCs induce effector or suppressor T cell responses will be determined by the balance between the levels of positive costimulatory factors (e.g. CD40, ICOSL, and CD86) and negative costimulatory molecules (e.g. PDL-1, and B7H3), and the levels of MHC class I and II molecules. The PDL-1 is expressed on monocytes and mature DCs and exerts inhibitory effects on CD4+ T cell activation [46], [47]. B7H3 is expressed in high levels on immature DCs and moderate levels on mature DCs [48]. We found that the negative costimulatory molecules PDL-1 and B7H3 were up regulated ex vivo on blood MDCs and PDCs from PDAC patients and this could be one reason for the impaired immunostimulatory capacity. The treatment of blood DCs purified from healthy donors with plasma from PDAC patients or CXCL8 and PGE_2_ gave similar effect, with significantly enhanced PDL-1 levels. We found increased expression of B7H3 on MDCs and PDCs ex vivo, in contrast to the rest of the markers that all followed the normal maturation profile induced by e.g. TLR ligands. CXCL8 and PGE_2_ treatment of purified MDCs and PDCs decreased these cells expression of B7H3, which followed the pattern seen for TLR stimulated DCs. The implication of enhanced B7H3 on MDCs and PDCs in PDAC needs to be further assessed as this costimulatory molecule has been shown to exert a negative effect on DCs' ability to activate T cells [49], [50], moreover, up regulation of B7H3 could be one mechanism whereby the tumor escapes from the immune system. Our results indicate that the PDAC patients with the best clinical outcome retained high expression of ICOSL on both MDCs and PDCs and CCR2 on PDCs, which indicate that the DCs were less affected by the tumor. Of note, none of the other factors examined correlated with survival.

The inflammatory factors PGE_2_ and CXCL8 did not achieve full maturation rather semi matured MDCs and PDCs, which was not a surprise seeing that full maturation, when induced by inflammatory factors, require a combination of factors such as TNF-α, PGE_2_, IL-6, and IL-1β [51]. PGE_2_ has previously been shown to induce upregulation of some maturation associated molecules, e.g. CCR7, but not to induce full maturation and may even induce the immune inhibitory molecule indoleamine 2,3 dioxygenase expression in DCs [52], [53]. CXCL8 is normally not considered as a maturation factor, rather as a chemotactic factor and exerted no or very low effect on the DC maturation,

DCIR belongs to the C-type lectin receptors and exerts immune inhibitory functions through its intracellular signaling motif, ITIM [18], DCIR is down modulated upon maturation [18] and this fits with our ex vivo data as both MDCs and PDCs had significantly decreased levels of this C-type lectin.

The mechanism creating these semi-mature DCs seems to be inflammatory factors in the blood and our study provides evidence for the involvement of CXCL8 and PGE_2_ in this process. As shown previously by us, the inflammatory profile in blood showed significant increased CXCL8 and PGE_2_ but also elevated levels of other factors, such as MCP-1, MIP-1b, and IP-10 in PDAC patients [14], that could be of relevance for the semi maturation of MDCs and PDCs seen ex vivo. The surgical removal of the tumor mass decreased the CXCL8 and PGE_2_ levels [14] but not to normal levels, which could explain why the partial DC maturation was not reversed. The source of the CXCL8 and PGE_2_ should be the pancreatic tumor microenvironment itself as very high levels of both CXCL8 and COX-2 are expressed at this site [54], [55] especially by the fibrotic stroma surrounding the tumor nests (Tjomsland et al work in progress).

In conclusion, in individuals with PDAC, blood MDCs and PDCs are impaired in number and exhibit a semi mature phenotype. These remaining DCs do not have the ability to function as professional antigen presenting cells, and this could be one part of the tumor suppression of the adaptive immune response, including the impaired T cell responses observed. Moreover, the sustained expression of ICOSL on both MDCs and PDCs and CCR2 on PDCs was a strong indicator of patient survival. We also showed that PGE_2_ and CXCL8 in collaboration with other inflammatory factors existing systemically in PDAC patients were responsible for the changes seen in the blood DCs, suggesting that clearing the inflammation in this disease may be beneficial for patient survival and quality of life.

## Methods

### Ethics Statement and subjects enrolled in the study

The study protocol and patient consent documents were approved by the Regional Ethics Committee in Linköping, Sweden (Dnr. M38-06). The consent was written and obtained from all participants involved in the study. Blood samples from 20 patients with PDAC, 5 patients with chronic pancreatitis, and 20 age matched controls were used in this study. Heparinized peripheral whole blood samples (20 ml) were obtained from controls, at one occasion, and from patients at two time points, one week prior surgical removal of the tumor (Whipple resection) and 8–12 weeks after the surgery at Linköping University Hospital (Linköping, Sweden). The age matched controls were recruited randomly from department of Transfusion Medicine at Linköping University Hospital and from the senior division of Linköping orienteering club. The PDAC patients did not receive any chemo/radiotherapy during the time period of the blood sample collection (i.e. pre or post surgery) and had no long term treatment with cortisone or NSAID. The PDACs were staged according to the 1997 International Union against Cancer classification (TNM = Tumor, Node, Metastasis) and the PDAC patients ranged from T2-T4, N0 (N = 5), N1 (N = 20) and M0 (N = 25) stage.

### Separation of peripheral blood mononuclear cells

Peripheral blood mononuclear cells (PBMCs) were isolated from heparin treated whole blood by Ficoll-Paque PLUS (GE Healthcare, Uppsala, Sweden) density gradient centrifugation. The plasma layer was collected after the density centrifugation, aliquoted and stored at −70°C until analysis. The cellular interface containing the PBMCs was harvested and washed twice in PBS (PAA Laboratories GmbH, Germany). The PBMCs were resuspended in freezing media (fetal bovine serum containing 8% DMSO (Sigma-Aldrich, Schnelldorf, Germany)) and cryo preserved until further analysis.

### Monoclonal antibodies for flow cytometry

Peripheral blood DC subsets were identified using Alexa Fluor 700 conjugated lineage (Lin) cocktail (CD3, CD14, CD16, CD19, CD20, and CD56; AbD Serotec, Germany), HLA DR (APC-Alexa Fluor 750), CD11c (PE-Cy7), and CD123 (PerCP-Cy5,5) monoclonal antibodies (mab) (eBioscience, CA, USA). Phenotypical characterization of MDCs and PDCs were executed using three different cocktails of antibodies, i.e. cocktail A consisting of CD275 (Biotin), CD40 (FITC) (eBioscience), and CD83 (APC) (Becton Dickinson, Stockholm, Sweden); B consisting of CD192 (Biotin) (R&D Systems, MN, USA), CD196 (FITC) (Biolegend), CD195 (PE) and CD197 (Alexa Fluor 647) (Becton Dickinson, Stockholm, Sweden); C consisting of DCIR (Biotin) (Dendritics, Lyon, France), CD276 (FITC) (AbD Serotec), CD273 (PE) (Becton Dickinson, Stockholm, Sweden) and CD274 (APC) (eBioscience).

### Flow cytometry acquisition and analysis

PBMCs (1×10^6^) were suspended in PBS supplemented with 0.2% BSA (FACS wash) and labeled with lineage cocktail, HLA DR, CD11c, and CD123 mAbs to detect MDCs and PDCs in addition to matching isotype controls and antibodies included in cocktail A–C (see above). The antibody incubation was carried out at 4°C for 60 min. After the incubation unbound antibodies were removed by spinning down the samples and replacing the supernatant with new FACS wash. This procedure was repeated 2 times. To detect the biotin marked antibodies, Streptavidin eFluor 450 (eBioscience) was added to the samples and incubated at 4°C for 15 min. Eight color flow cytometry was performed using a FACS ARIA flow cytometer (Becton Dickinson, San Jose, CA), analyzing 5×10^5^ PBMCs. The acquired data was analyzed using the FLOW-JO software, v7.0 (Tree Star Inc, Ashland, OR).

### MDC and PDC activation of allogeneic T cells

MDCs and PDCs were purified from PBMCs derived from PDAC patients or healthy donors by depletion of CD19 positive cells, followed by positive selection of MDCs using BDCA-1 microbeads and PDCs using BDCA-4 microbeads (Miltenyi Biotec). Mixed lymphocyte reaction was performed by plating MDCs and PDCs together with allogeneic T cells in a 96-well pate at ratios of 1:10–1:30. To maintain healthy DCs throughout the experiment, PDCs were cultured in the presence of 10 ng/ml of IL-3 (Sigma-Aldrich) and MDC with GM-CSF (100 IU/ml) (Genezyme). The MLR was cultured for 5 days and pulsed with 1 µCi ^3^H-Thymidine during the last 12 h of the culture period. The assay was harvested and the incorporated radioactivity counted in a β-counter. The immunostimulatory activity was compared between PDAC patients and healthy controls by dividing the counts per minute (CPM) of patients with the mean CPM of controls and the CPM of the controls with the mean CPM of the patients for each plate analyzed.

### Sorting of MDCs and PDCs from healthy donors

PBMCs were isolated from 450 ml heparin treated whole blood received from randomly recruited donors at the Department of Transfusion Medicine, Linköping University Hospital (Linköping, Sweden). The PBMCs were labeled with CD3, CD14, CD19 and CD56 microbeads for depletion of T cells, B cells, NK cells and monocytes according to manufactures description (Miltenyi Biotec, Germany). The remaining cells were incubated at 4°C for 60 min with lineage cocktail (Alexa Fluor 700), HLA DR (APC-Alexa Fluor 750), CD11c (PE-Cy7) and CD123 (PerCP-Cy5.5) mabs, washed and run through a pre separation filter (Miltenyi Biotec) and sorted into MDC and PDC populations using a FACS ARIA cell sorter.

### Assessment of effects of PDAC patient plasma and CXCL8 and PGE2 on purified blood DCs from healthy donors

Pure populations of MDCs and PDCs (6.5×10^4^ cell/well), were exposed to 10 ng/ml of CXCL8 and PGE_2_, as single or double agents over night (24 h). One percent plasma medium (RPMI 1640 (Fisher Scientific, Pittsburgh, PA)) with or without maturation stimulatory factors, Poly I:C (MDC) and C CpG (Sigma-Aldrich, Sweden) (PDC), were used as experimental controls. Single patient and control plasma or pooled plasma from 6 randomly selected patients and controls, respectively, were added to 6.5×10^4^ MDCs or PDCs in a 1:4 dilution of plasma. After 24 h of culture, the DCs were stained with the same lines of antibodies as described earlier (cocktail A, B, and C) and analyzed using a FACS ARIA flow cytometer, analyzing 5×10^3^ DCs. The acquired data was analyzed using the FLOW-JO software, v7.0 (Tree Star Inc, Ashland, OR).

### Statistics

The statistical analysis was performed using GraphPad Prism 5 (GraphPad Software, La Jolla, CA). A p-value of <0.05 was considered statistically significant and error bars throughout indicate standard error of the mean (SEM). Non-parametric data was analyzed using the Wilcoxon matched pairs test followed by Mann-Whitney test and Paired t-test was used for normalized data. Survival curves were analyzed by the Kaplan-Meier survival method, and statistical significance was determined using Log-rank (Mantel-Cox) test and a p value <0.05 was considered statistically significant.
